# Intraoperative Extravascular Ultrasound in the Identification of Flow-Limiting Dissections after Balloon Angioplasty in the Femoropopliteal Segment

**DOI:** 10.3390/jcm13061635

**Published:** 2024-03-13

**Authors:** Bibombe Patrice Mwipatayi, James Dodd, Joseph Hanna, Yann Gouëffic, Marianne Brodmann, Mercedes Guerra, Andrej Schmidt, Christian Loewe, Gerd Grözinger, Grigorios Korosoglou, Michael Lichtenberg, Koen Deloose

**Affiliations:** 1 Department of Vascular Surgery, Royal Perth Hospital, Perth 6000, Australia; james.dodd@health.wa.gov.au (J.D.); joseph.hanna@health.wa.gov.au (J.H.); 2 Department of Vascular and Endovascular Surgery, Groupe Hospitalier Paris St. Joseph, 75014 Paris, France; ygoueffic@ghpsj.fr; 3 Department of Angiology and Vascular Medicine, Medical University Graz, 8010 Graz, Austria; marianne.brodmann@medunigraz.at; 4 Vascular and Endovascular Surgery Department, University Hospital of Guadalajara, 19002 Madrid, Spain; mguerrar@sescam.jccm.es; 5 Department of Interventional Angiology, University Hospital Leipzig, 04103 Leipzig, Germany; andrej.schmidt@medizin.uni-leipzig.de; 6 Department of Bioimaging and Image-Guided Therapy, Division of Cardiovascular and Interventional Radiology, Medical University of Vienna, 1090 Vienna, Austria; christian.loewe@meduniwien.ac.at; 7 Abteilung für Diagnostische und Interventionelle Radiologie, University Hospital Tübingen, 72076 Tübingen, Germany; gerd.groezinger@med.uni-tuebingen.de; 8 Cardiology and Vascular Medicine, GRN Hospital Weinheim, 69469 Weinheim, Germany; gkorosoglou@hotmail.com; 9 Vascular Center, Klinikum Arnsberg, 59821 Arnsberg, Germany; klichte@gmx.net; 10 Vascular Surgery, AZ Sint Blasius Hospital, 9200 Dendermonde, Belgium; koen.deloose@vascular.be

**Keywords:** extravascular ultrasound, flow-limiting dissection, drug-coated balloon, femoropopliteal artery

## Abstract

**Background:** The BIO REACT study is designed to investigate the incremental value of Extravascular UltraSound (EVUS) added to conventional angiography, compared to conventional angiography only for the identification of Flow-Limiting Dissections (FLD) and to evaluate the safety and efficacy of the REsponse Adapted Combination Therapy (REACT) for the treatment of femoropopliteal lesions. **Methods:** The primary endpoints were the specificity and sensitivity of EVUS added to angiography for the detection of FLD. Secondary endpoints were primary patency of the REACT therapy within 12 months, fCD-TLR, freedom from MAE, major target limb amputations (mTLA) and survival rates within 24 months. **Results:** A total of 150 patients were included. EVUS added to angiography had an overall sensitivity of 29% and specificity of 93% for the detection of FLD. There was no PSVR cut-off offering a clinically acceptable trade-off between meaningful sensitivity and specificity values for the detection of FLD. At 12 months, treatment with the REACT resulted in primary patency and fCD-TLR of 81.6% and 94.3%, respectively. In addition, freedom from MAE was 94.3% at 12 months. At 24 months, the survival rate was 94.0%. No mTLA was reported up to the 24-month follow-up. **Conclusions:** The addition of DUS to angiography showed limited value for detecting FLD in femoropopliteal artery disease.

## 1. Introduction

Due to recent technical advances, drug-coated balloon angioplasty (DCBs) has currently emerged as a first-line option for the treatment of atherosclerotic femoropopliteal disease [1]. DCBs reduce vessel restenosis due to the release of an antiproliferative drug into the vessel. Moreover, since a permanent scaffold (stent) is not left within the lesion, all surgical and endovascular intervention options remain for future intervention. In more complex lesions, stent placement may be required for the management of flow-limiting dissection (FLD) or significant residual stenosis or recoil [2,3,4].

REsponse Adapted Combination Therapy (REACT) is a treatment approach aiming at minimizing the metal burden associated with stent placement and subsequent stent-related complications. It combines the Passeo-18 Lux DCB (Biotronik AG, Bülach, Switzerland) as a primary treatment strategy and, if required, by bail-out stenting using the Pulsar-18 or Pulsar-18 T3 thin strut self-expandable stents (Biotronik AG, Bülach, Switzerland), though only if necessary.

For the selection of lesions that require stent placement after DCB, an angiography-only strategy may not always provide adequate detail for the detection of FLD [5]. Thus, alternative diagnostic tests may become necessary. In this regard, Extravascular Ultrasound (EVUS) is the diagnostic method of choice to identify significant stenosis in the lower extremity arteries. Although it may be more challenging in infrapopliteal arteries [6,7,8], it is non-invasive, cost-effective, accessible and widely used for diagnosis and post-revascularization follow-up.

The REsponse Adapted Combination Therapy Pilot Study (BIO REACT) was designed to (i) investigate the incremental value of intraoperative EVUS added to conventional angiography compared to conventional angiography only for the identification of FLD and (ii) to evaluate the clinical performance and safety of Passeo-18 Lux DCB alone or combined with Pulsar-18 or Pulsar 18 T3 stent for the treatment of de novo or restenotic femoropopliteal lesions.

## 2. Materials and Methods

### 2.1. Study Design and Patients

BIO REACT was a prospective, multicentre, pilot diagnostic study (ClinicalTrials.gov: NCT03547986) with follow-up visits at 1, 6, 12 and 24 months. This study was conducted in compliance with the International Conference on Harmonization Good Clinical Practice, ISO 14155 [9], and the Declaration of Helsinki. The local ethics committees at the participating sites approved the study protocol, and all participants provided written informed consent before any study-specific test or procedure.

Eligible participants were ≥18 years of age with chronic, symptomatic lower limb ischemia as defined by Rutherford Category (RC) 2 to 4. Angiographic criteria for enrolment included atherosclerotic lesions (de novo, restenotic or [re]occluded lesion[s] post percutaneous transluminal angioplasty [PTA]) with ≥70% diameter stenosis by visual angiographic assessment located in the native superficial femoral artery (SFA) and or proximal popliteal artery (PPA). Reference vessel diameter was required to be between 4.0 and 6.0 mm.

Key exclusion criteria were target lesion previously treated with DCB < 12 months prior to enrolment; previously stented target lesion; use of atherectomy, laser or other debulking devices in the target SFA/PPA vessel during the index procedure; prior or planned major target limb amputation (mTLA—defined as any lower limb amputation above the ankle); presence of aneurysm in the target vessel; uncorrected bleeding disorder; presence of renal failure; and acute ischemia and/or acute thrombosis of the target SFA/PPA vessel prior to enrolment.

### 2.2. Study Devices

Passeo-18 Lux DCB (Biotronik AG, Bülach, Switzerland) is indicated for dilatation of lesions in the infrainguinal arteries with simultaneous release of paclitaxel (3 µg/mm^2^) to the vessel wall to reduce the occurrence of restenosis of the treated vessel segment. Both Pulsar-18 and Pulsar-18 T3 are self-expanding stent systems indicated for patients with atherosclerotic disease of the femoral and infrapopliteal arteries and for the treatment of insufficient results after PTA, e.g., residual stenosis and dissection.

### 2.3. Study Procedures

Procedural techniques were performed according to the hospital standard of care for endovascular treatment of the femoropopliteal segment. A baseline diagnostic angiogram of the whole limb was performed prior to dilatation to characterize the target lesion and confirm remaining procedure-related eligibility criteria. Pre-dilatation was then performed with the Passeo-18 (Biotronik AG, Bülach, Switzerland) PTA balloon progressively inflated for at least 3 min. Consistent with the REACT approach, following successful pre-dilatation, the target lesion was treated with Passeo-18 Lux DCB. To maximize the mechanical effect and drug uptake, the DCB was inflated gradually, and the inflation was maintained for at least 3 min. In the case of a complication occurring during vessel preparation that, in the opinion of the investigator, would have prevented the lesion treatment according to REACT algorithm, the patient was not enrolled. After target lesion dilatation with the DCB, an angiogram was performed in at least two planes. The lesion was then evaluated by EVUS, and stenting of the lesion was performed at the operator’s discretion with a self-expanding stent. Finally, a completion angiogram was performed to allow for the assessment of residual stenosis and vessel dissection pattern. The decision on antiplatelet therapy was left at the discretion of the attending physicians based on local practice and standard operating procedures of the corresponding site.

### 2.4. Intraoperative Extravascular Ultrasound

It is generally accepted that a peak systolic velocity ratio (PSVR) of more than 2.5 is related to angiographically significant arterial stenosis of greater than 50% [10,11,12]. However, the identification of flow-limiting dissection by EVUS relies on a combination of morphologic and hemodynamic assessments. In this study, a morphological assessment in B-mode beginning in the distal common femoral artery and extending to the native vessel segment distal to the target lesion was performed first. Then, the Doppler signal was measured at a 60° insonation angle to the vessel centreline along the same vessel segment. The peak systolic velocity (PSV) was measured proximal to the target lesion (PSVprox) at the level of the dissection or the largest degree of residual stenosis identified by two-plane angiography and distal to the target lesion (PSVdist). The final treatment decision for stent placement was guided by angiography and EVUS findings.

### 2.5. Endpoints and Definitions

The primary endpoint of the study was the diagnosis of flow-limiting dissection. This endpoint was used to examine specificity and sensitivity of EVUS added to angiography for various peak systolic velocity ratio (PSVR) values. A receiver operating characteristic (ROC) curve was plotted to assess the test accuracy and optimal PSVR cut-off values. Angiographic data were obtained by the investigator at the time of the index procedure. Angiograms were reviewed separately by an Independent Review Committee (IRC) consisting of two experienced endovascular operators blinded to EVUS and clinical data. Interpretation disagreements were resolved by consensus. The angiography analysed by the IRC was used as the standard diagnostic reference.

Secondary procedural endpoints included the target lesion stenting rate, number of stents used and mean stent length per target lesion, mean target lesion length stented (full, spot), DCB technical success (defined as the delivery and successful use of the DCB to the target lesion to achieve residual stenosis of less than 30% in the absence of FLD), stent technical success (defined as the delivery and successful use of the self-expanding stent to the target lesion to achieve residual stenosis of less than 30%) and procedural success (defined as the technical success and no major adverse events (MAE) before discharge).

Secondary clinical performance endpoints included the primary patency (defined as PSVR ≤ 2.5m/s in the target lesion, in the absence of clinically driven target lesion revascularization [CD-TLR]) and CD-TLR rate at 1, 6, 12 and 24 months. Secondary safety endpoints included the MAE rate (defined as device- or procedure-related death within 30 days post index procedure, major target limb amputation or CD-TLR post index procedure), major adverse limb event (MALE) rate (defined as severe limb ischemia leading to an intervention or mTLA), mTLA rate and death rate (all causes) at 1, 6, 12 and 24 months.

### 2.6. Statistical Analysis

Descriptive statistics were used to analyse the data. Quantitative variables were reported as the mean values with standard deviation and two-sided 95% CI (confidence interval). Qualitative variables were reported as absolute and relative frequencies with exact Binomial two-sided 95% CI. Sensitivity and specificity of angiography with and without EVUS were calculated. A ROC curve was plotted to determine the optimal PSVR cut-off for detecting FLD. Primary patency, freedom from MAE, freedom from CD-TLR, freedom from major target limb amputation, freedom from MALE and survival were estimated by the Kaplan–Meier analyses. Estimates were presented with 95% CI. All statistical analyses were performed using the SAS statistical package, version 9.4 (SAS Institute, Cary, NC, USA).

## 3. Results

### 3.1. Patient and Lesion Characteristics

A total of 150 patients at 15 clinical sites in Europe and Australia were included in the study. The mean age was 68.7 ± 9.8 years, and 102 (68%) were men. Most patients had claudication (RC 2 or 3, 87.8%), while the remaining had ischemic rest pain (RC 4, 12.2%). Most patients had hypertension (80%), were current or ex-smokers (80%), had hyperlipidaemia (74%) and a history of peripheral arterial occlusive disease (59.3%). The baseline demographic data and clinical characteristics are summarized in Table 1.

A total of 167 lesions, with a mean length of 103.3 ± 73.4 mm and diameter stenosis of 89.0 ± 11.1% according to visual estimate, were treated. The mean reference vessel diameter was 5.2 ± 0.7 mm. Of the 167 treated lesions, 85% were found in the SFA, 11.4% of lesions were extending from SFA to the proximal popliteal artery, and 3.6% were in the proximal popliteal segment. Additionally, most target lesions were either de novo stenotic lesions (60.5%) or occlusions (34.7%). Restenosis and re-occlusions occurred in 3.0% and 1.8% of cases, respectively. Most of the lesions were calcified (76%), with 27.6% moderately to heavily calcified. Trans-Atlantic Inter-Society Consensus (TASC) lesion classification as either A, B, C or D was 28.7%, 39.5%, 24.0% and 7.8%, respectively. Lesion characteristics are summarized in Table 2.

### 3.2. Procedural Characteristics

The mean procedure duration was 92.9 ± 42.5 min. The preferred access site was the femoral artery (99.3%). A 6 Fr access sheath (66.7%), crossover direction (54.7%) and intraluminal approach (94.0%) was used. Target lesion preparation with Passeo-18 was performed for 98.2% of lesions, often using one balloon (87.4%). The target lesion was treated with either one (62.3%), two (24.0%) or three (13.2%) DCBs. Residual stenosis post DCB treatment (visual estimate upon angiography) was 22.4 ± 21.9%. A total of 75 (44.9%) post-DCB dissections were reported, of which 26 (34.7%) were considered FLD. According to the IRC consensus data, there were a total of 98 (67.6%) dissections, of which 42 (42.9%) cases were considered FLD. Post-dilatation with plain old balloon angioplasty (POBA) occurred in 41.9% of the cases. The target lesion treatment outcomes are summarized in Table 3. Post-treatment with stent placement using a self-expanding stent was performed in 35.3% of cases with a mean length of 98.1 ± 45.3 mm. The technical success was 95.4% for DCB and 98.3% in case of provisional stenting. Procedure success was achieved in 144 of 150 patients (96.0%). Procedural characteristics are summarized in Table 4.

### 3.3. Diagnostic Performance of EVUS Added to Angiography for Detection of FLD

**Figure 1:**
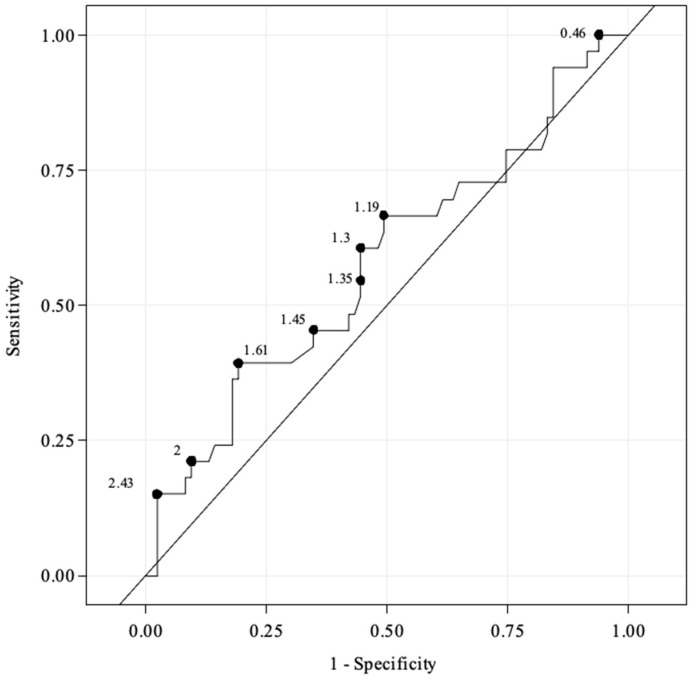
Receiver operating characteristic curve for peak systolic velocity ratio values according to flow-limiting dissection status based on Independent Review Committee angiography data.

Of the 167 treated lesions, 121 (72%) lesions had both angiography and EVUS available and were evaluable with a clear dissection status (FLD or no FLD). Compared to IRC angiography, angiography with additional EVUS had an overall sensitivity of 29% (95% CI, 14.6–46.3) and specificity of 93% (95% CI, 85.4–97.4) (Table 5). Receiver operating characteristic curves demonstrated poor prediction of FLD based on PSVR values. The exact numerical values from the ROC curve are shown in Figure 1. There was no PSVR cut-off offering a clinically meaningful trade-off between sensitivity and specificity to detect FLD.

### 3.4. Efficacy and Safety Outcomes of the Combination Therapy

The 12-month Kaplan–Meier primary patency estimate was 81.6% (95% CI, 73.6–87.4) (Figure S1). The freedom from CD-TLR by a Kaplan–Meier estimate was 94.3% (95% CI, 89.3–97.0) at 12 months and 87.7% (95% CI, 81.0–92.1) at 24 months (Figure S2). The freedom from MAE was 94.3% (95% CI, 88.9–97.1) and 87.7% (95% CI, 80.5–92.3) at 12 and 24 months, respectively (Figure S3). The freedom from MALE by a Kaplan–Meier estimate was 92.4% (95% CI, 86.4–95.7) and 88.6% (95% CI, 82.0–92.8) at 12 and 24 months, respectively (Figure S4). No mTLA was reported through the 24 months of follow-up. The survival rate was 97.2% (95% CI, 92.8–99.0) at 12 months and 94.0% (95% CI, 88.2–97.0) at 24 months (Figure S5). A total of eight deaths occurred during the 24-month follow-up period. However, the Clinical Events Committee (CEC) did not attribute any of these deaths to the device or procedure.

## 4. Discussion

BIO REACT pilot study is the first exploratory study designed to evaluate the potential benefits of using intraoperative EVUS alongside angiography to detect FLD. Currently, dissections are a common occurrence during femoropopliteal arterial interventions [13]. However, there is still no reliable methodology that can be easily used in clinical practice to help clinicians identify and grade dissections. This lack of reliable methodology makes it difficult to guide treatment strategies.

Although the National Heart, Lung, and Blood Institute’s classification system has been widely used to grade coronary artery dissection based on angiographic features, it cannot be applied to peripheral arteries [14]. The incongruity of femoropopliteal arteries and coronary vessels arises from substantial variations in their characteristics, including vessel length and diameter, as well as disease burden. Despite its frequent application in clinical trials to grade dissection severity, this categorization has yet to be widely adopted in daily practice. This led to another angiographic categorization system, which was proposed by Kobayashi et al. [15]. The Kobayashi classification system based on angiography comprises three primary categories: no angiographic dissection, mild dissection (where the dissection width is less than one-third of the lumen diameter) and severe dissection (where the dissection width is more than one-third of the lumen diameter). It is a simple classification system with a higher potential for translation into clinical practice [15].

Previous studies based on the visual evaluation of dissections after angioplasty reported increasing rates of bail-out stenting with increasing dissection severity by angiographic criteria [16]. However, to improve the evaluation of dissection, it is worth considering supplementing the current angiographic dissection classification systems with additional methods like intravascular ultrasound (IVUS) or EVUS [5]. Given that IVUS provides more sensitive and quantitative data on the grade and extent of dissections, it could be a valuable tool for the detection and effective management of dissections [5,17]. The IVUS-based classification system could be an alternative as it provides information that may accurately determine both the depth and degree of arterial dissection. The recently published iDissection grading system was developed for this purpose [18]. It consists of six grades of dissection based on the depth and circumference of injury as revealed on IVUS. Although this system provides a framework to classify the degree and extent of dissection, it fails to consider the dissection length and, importantly, blood flow. More recently, the DISFORM classification system was developed through an expert panel as a tool to standardize reporting for peripheral arterial dissections [19]. While this system offers relative simplicity and intuitiveness in differentiating dissection severity and related outcomes, it remains a descriptive tool based on subjective assessment. The external validity of this classification needs to be evaluated in a real clinical setting. Intravascular ultrasound-based systems remain the most accurate way of identifying dissections and provide greater detail than angiography. However, IVUS use is limited by availability and operator experience. In addition, the reimbursement of IVUS is not provided in many European countries, limiting its use within clinical studies and specialized centres.

Although EVUS is widely adopted as a diagnostic and follow-up method for lower extremity peripheral arterial disease, intraoperative EVUS use is not clinically established. Few articles report on the EVUS-guided endovascular treatment of lower limb arteries [20,21,22], and none on the adjunctive values of EVUS confirm angiographic findings. Extravascular Ultrasound, like IVUS, provides a more precise assessment of arterial diameter compared to angiography. We hypothesised that the addition of EVUS would provide additional information for clinicians with respect to haemodynamic, residual stenoses and dissection. However, this pilot study found that EVUS has limited additional value in identifying FLD in the treatment of femoropopliteal lesions compared to angiography alone. In the detection of femoropopliteal dissections, angiography and EVUS combined had a sensitivity of 29% and a specificity of 93%. The rationale underlying this is likely multifactorial, including technical and operator-related factors. Given intraoperative EVUS is not standard practice, a lack of familiarity with how to interpret intraoperative haemodynamic changes on EVUS consistent with dissection may have been a contributing factor toward its low sensitivity in this study. Recently, Fazzini et al. [23] proposed a new duplex-assisted protocol for the endovascular treatment of lower limb artery lesions which, if widely adopted, could enable physicians to better appreciate the EVUS-detected haemodynamic changes associated with FLD.

The BIO REACT pilot study findings suggest variations in the rates of FLD identified by angiography, as reported by investigators and IRC reviewers. Specifically, based on angiography paired data (*n* = 121), dissections categorised as flow-limiting were considered in 28.9% by IRC reviewers and 16.5% by the investigators. This observation is consistent with the TOBA study [24], in which operators reported severe dissection (grade ≥ C) in 25.8% of patients, whereas an independent core laboratory analysis reported grades ≥ C in 74% of patients. This result demonstrates that FLDs are underreported and that the severity of identified FLDs may be underestimated. Therefore, less FLD lesions are treated optimally. Operators should be cognisant of this and maintain a high index of suspicion for FLD intraoperatively to minimise dissection-related complications. The literature suggests IVUS remains a useful adjunct to assist with the detection of FLD.

Although stent implantation could not be significantly reduced with the use of EVUS, the results of the BIO REACT pilot study show the safety and clinical performance of the REACT approach to treat lesions in the femoropopliteal segment. In our study, the survival rate was 94.0% (95% CI, 88.2–97.0) at 24 months, and there were no major reported amputations of the target limb. The technical success rates were high at 95.4% for DCB and 98.3% for provisional stenting, while the procedural success rate was 96.0%.

This study has several limitations. Despite the use of angiography in two orthogonal planes with an examination by independent reviewers that may serve as an objective measure of FLD, this method cannot be considered a gold standard as it misses several patterns of dissections. Since there is currently no available standardized definition of FLD in the literature to date, no definition was provided for the study. The degree of impairment of the blood flow post angioplasty was evaluated by investigators and independent reviewers’ visual assessment. Also, it must be recognised this finding has limited generalisability as this study assessed only femoropopliteal lesions in a limited number of patients. Additionally, despite diligent efforts to ensure compliance with the angiography process, the considerable variability observed between centres in angiographic imaging quality had a direct impact on the reduced number of cases available for analysis by the IRC. Finally, as the study was performed under routine clinical practice, investigators were not trained to capture morphologic and hemodynamic EVUS parameters that could predict FLD. Future investigation could consider repeating this study after additional EVUS training for operators with an emphasis on identifying FLD.

## 5. Conclusions

In this exploratory diagnostic study, EVUS was shown to have limited added value when combined with angiography to detect FLD post-DCB angioplasty in femoropopliteal lesions. A further investigation of intraoperative EVUS associated with pre- and acute post-operative DUS assessment performed by trained operators following predefined protocol is needed to determine morphologic and hemodynamic parameters that are predictors of FLD. The REsponse Adapted Combination Therapy combining Passeo-18 Lux DCB and Pulsar-18 or Pulsar-18 T3 stent when and where needed was shown to be safe and effective.

## Figures and Tables

**Table 1:** Baseline patient demographic and clinical characteristics.

Description	Total (*n* = 150)
Age (yrs.)	68.7 ± 9.8
Male	102 (68.0%)
Female	48 (32.0%)
Hypertension	120 (80.0%)
Ex- or current smoker	120 (80.0%)
Hyperlipidaemia	111 (74.0%)
History Peripheral Arterial Occlusive Disease	89 (59.3%)
Previous peripheral interventions/surgeries	57 (38.0%)
Coronary artery disease	46 (30.7%)
Diabetes	45 (30.0%)
Insulin-dependent	21 (14.0%)
Non-insulin-dependent	24 (16.0%)
Renal disease (insufficiency)	27 (18.0%)
Cerebrovascular disease	22 (14.7%)
Cancer	21 (14.0%)
Rutherford clinical category (*n* = 148)	
2	26 (17.6%)
3	104 (70.3%)
4	18 (12.2%)
Ankle–brachial index of the target limb	0.67 ± 0.16

**Table 2:** Baseline lesion characteristics.

Description	Total (*n* = 167)
Target lesion length (mm)	103.3 ± 73.4
Length of occluded section (mm)	89.6 ± 73.5
Reference vessel diameter (mm)	5.2 ± 0.7
Diameter stenosis (mm)	89.0± 11.1
Target lesion location	
PPA	6 (3.6%)
SFA	142 (85.0%)
SFA and PPA	19 (11.4%)
Target lesion type	
De novo stenotic lesion	101 (60.5%)
Restenosis	5 (3.0%)
Occlusion	58 (34.7%)
Re-occlusion	3 (1.8%)
Calcification scoring	
PACSS Grade 0	40 (24.0%)
PACSS Grade 1	36 (21.6%)
PACSS Grade 2	45 (26.9%)
PACSS Grade 3	22 (13.2%)
PACSS Grade 4	24 (14.4%)
TASC classification	
TASC A	48 (28.7%)
TASC B	66 (39.5%)
TASC C	40 (24.0%)
TASC D	13 (7.8%)

SFA: Superficial femoral artery, PPA: proximal popliteal artery, TASC: Trans-Atlantic Inter-Society Consensus document, PACSS: peripheral arterial calcium scoring system.

**Table 3:** Target lesion treatment.

Description	Total (*n* = 167)
Number of PTA balloons used	164 (98.2%)
1	146 (87.4%)
2	10 (6.0%)
3	6 (3.6%)
4	0 (0.0%)
5	1 (0.6%)
6	0 (0.0%)
7	1 (0.6%)
Number of DCBs used	167 (100%)
1	104 (62.3%)
2	40 (24.0%)
3	22 (13.2%)
4	1 (0.6%)
Number of stents used	59 (35.3%)
0	108 (64.7%)
1	46 (27.5%)
2	9 (5.4%)
3	4 (2.4%)
Length of stent (*n*= 76, mm)	98.1 ± 45.3
Post dilatation with POBA	70 (41.9%)
Number of POBA used	
1	66 (94.3)
2	4 (5.7%)
Target lesion length (*n* = 59 mm) for lesions treated with at least one stent	129.1 ± 77.1

**Table 4:** Procedural characteristics.

Description	Total (*n* = 150)
Procedure duration (min)	92.9 ± 42.5
Puncture site	
Femoral	149 (99.3%)
Pedal	1 (0.7%)
Direction	
Antegrade	59 (39.3%)
Crossover	82 (54.7%)
Retrograde	9 (6.0%)
Approach	
Sub-intimal	9 (6.0%)
Intraluminal	141 (94.0%)
Access sheath (French)	
4	12 (8.0%)
5	28 (18.7%)
6	100 (66.7%)
7	9 (6.0%)
8	1 (0.7%)
Residual stenosis post-DCB	22.4 ± 21.8
Dissection post-DCB (total) *	75 (44.9%)
Cat. A	24 (32.0%)
Cat. B	30 (40.0%)
Cat. C	9 (12.0%)
Cat. D	11 (14.7%)
Cat. E	0 (0.0%)
Cat. F	1 (1.3%)
Flow-limiting dissection post-DCB	26 (34.7%)
Post-procedural residual stenosis (%)	10.2 ± 10.4
Stenting rate	59 (35.3%)
DCB technical success **	103 (95.4%)
Stent technical success	58 (98.3%)
Procedural success	143 (95.3%)

* Dissection grading (NHLBI): Cat. A (minor radiolucent areas within the lumen during contrast injection with little or no persistence of contrast after the dye has cleared); Cat. B (parallel tracts or a double lumen separated by a radiolucent area during contrast injection, with minimal or no persistence after dye clearance); Cat. C (contrast outside the lumen (“extraluminal cap”) with persistence of contrast after dye has cleared from the lumen); Cat. D (spiral (“barber shop pole”) luminal filling defects, frequently with excessive contrast staining of the dissected false lumen); Cat. E (new, persistent filling defects within the lumen); Cat. F (lead to total occlusion of the lumen without distal antegrade flow). ** Lesion level not treated with stent.

**Table 5:** Diagnostic Performance of operator’ angiography with and without additional EVUS’ compared to IRC angiography alone.

Before EVUS		
	Angiography independent review ‘FLD’	Angiography independent review ‘No FLD’
Investigator reported upon ‘Angiography’: ‘FLD’	13 (11%)	7 (6%)
Investigator reported upon ‘Angiography’: ‘No FLD’	22 (18%)	79 (65%)
	Sensitivity 37% [21.5–55.1]	Specificity 92% [83.9–96.7]
**After EVUS**		
	Angiography independent review ‘FLD’	Angiography independent review ‘No FLD’
Investigator reported upon ‘Angiography + EVUS’: ‘FLD’	10 (8%)	6 (5%)
Investigator reported upon ‘Angiography + EVUS’: ‘No FLD’	25 (21%)	80 (66%)
	Sensitivity 29% [14.6–46.3]	Specificity 93% [85.4–97.4]

## Data Availability

Code and extracted data are available upon request.

## Supplementary Materials

The following supporting information can be downloaded at: https://www.mdpi.com/article/10.3390/jcm13061635/s1, Figure S1. Kaplan–Meier estimates for primary patency over 12 months (investigator-reported). Figure S2. Kaplan–Meier curve for freedom from clinically driven target lesion revascularisation over 24 months. Figure S3. Kaplan–Meier curve for freedom from major adverse events over 24 months. Figure S4. Kaplan–Meier curve for freedom from major adverse limb events over 24 months. Figure S5. Kaplan–Meier curve for survival rate over 24 months.
