# Supplementary material for: Intraoperative Extravascular Ultrasound in the Identification of Flow-Limiting Dissections after Balloon Angioplasty in the Femoropopliteal Segment
Source: J Clin Med. 2024 Mar 13;13(6):1635. doi: 10.3390/jcm13061635 (PMC10971548; doi:10.3390/jcm13061635)
Supplement: Supplementary file 1 [file jcm-13-01635-s001.zip › jcm-2873089-supplementary.pdf]

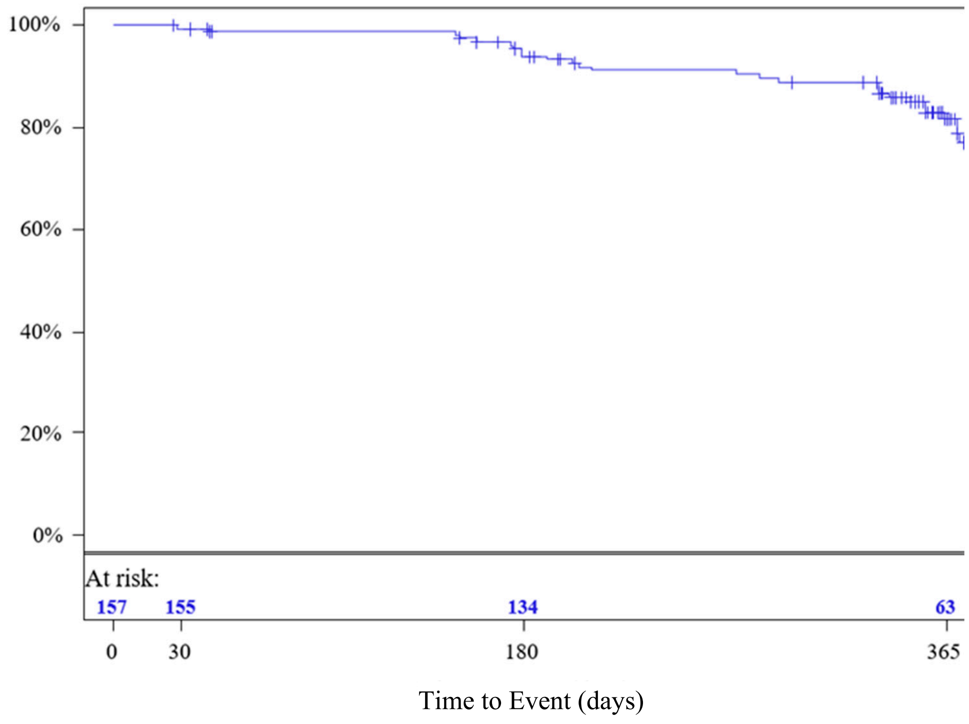

Description	Day 0	Day 30	Day 180	Day 365
At risk	157	155	134	63
Events	0	1	9	24
censored	0	1	14	73
Survival (estimate)	100.0 [100.0,100.0]	99.4 [95.5, 99.9]	93.9 [88.6, 96.8]	81.6 [73.6, 87.4]
SE	0.00000	0.00639	0.01966	0.03484

Figure S1. Kaplan-Meier estimates for primary patency over 12 months (investigator-reported).

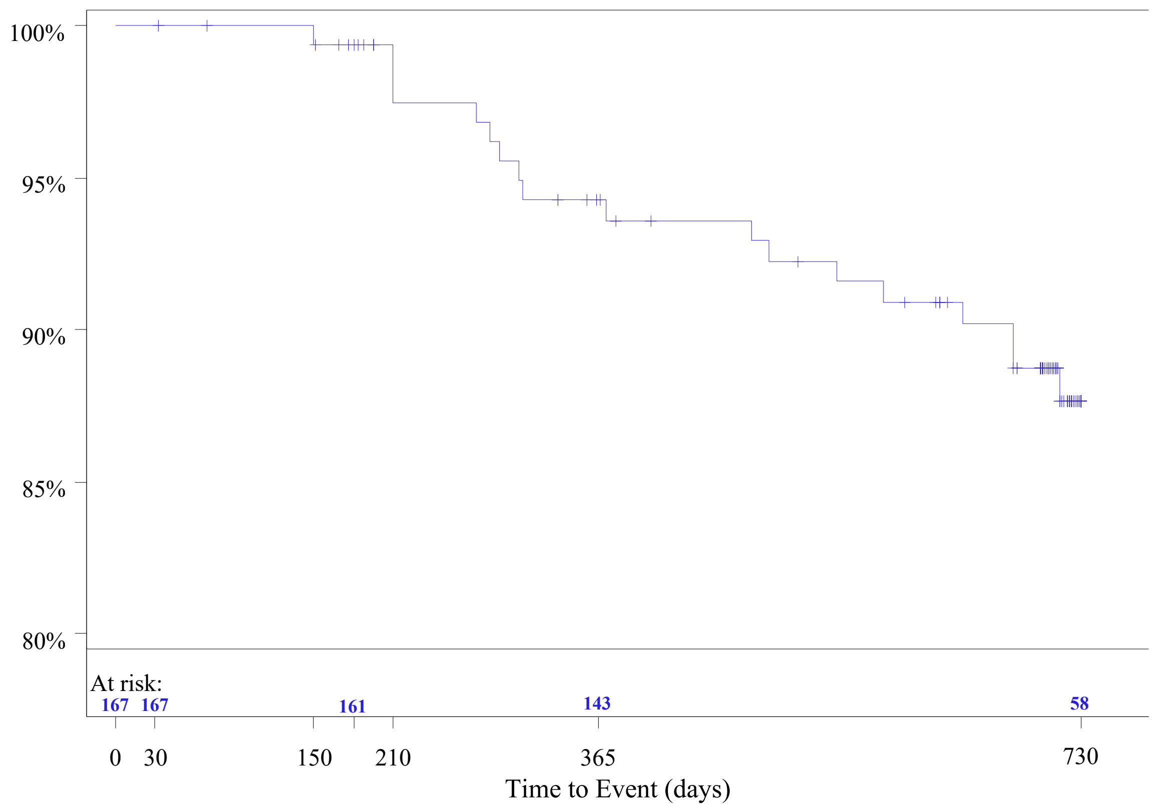

Description	Day 0	Day 30	Day 180	Day 365	Day 730
At risk	167	167	161	143	58
Events	0	0	1	9	18
Censored	0	0	5	15	93
Survival (estimate)	100.0 [100.0,100.0]	100.0 [100.0,100.0]	99.4 [95.8, 99.9]	94.3 [89.3, 97.0]	87.7 [81.0, 92.1]
SE	0.00000	0.00000	0.00604	0.01857	0.02770

Figure S2. Kaplan Meier curve for freedom from clinically driven target lesion revascularisation over 24 months.

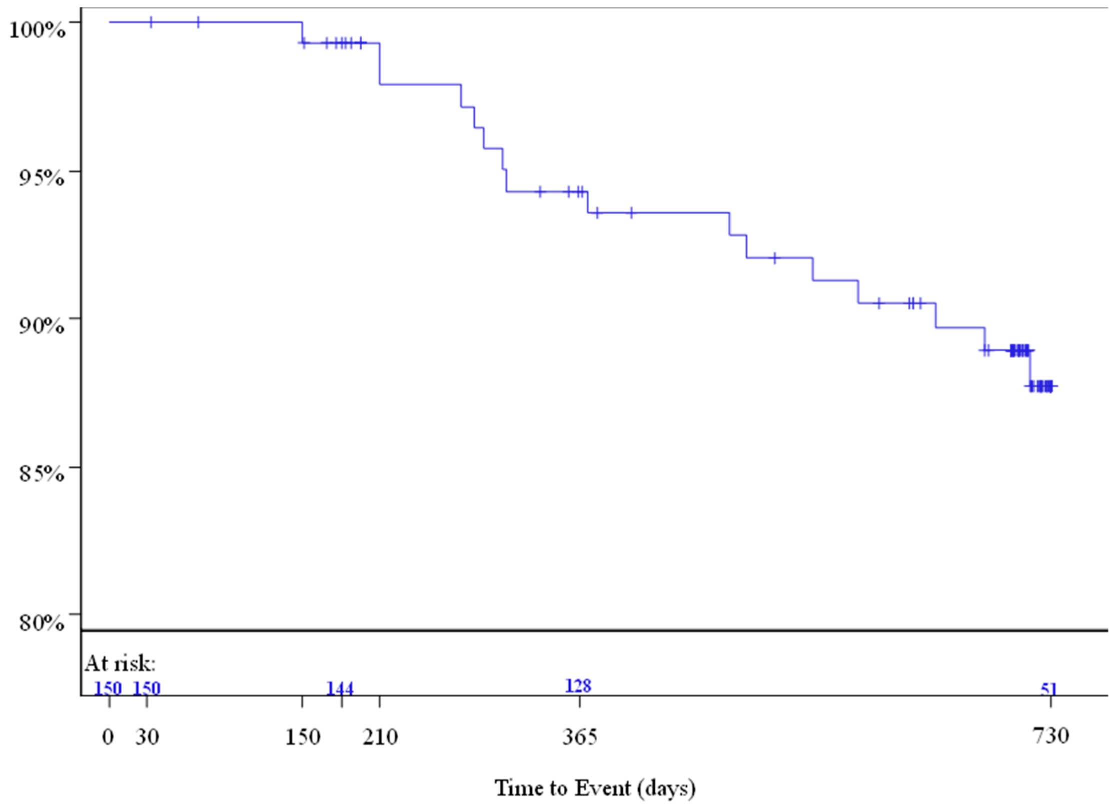

Description	Day 0	Day 30	Day 180	Day 365	Day 730
At risk	150	150	144	128	51
Events	0	0	1	8	16
Censored	0	0	5	14	85
Survival (estimate)	100.0 [100.0,100.0]	100.0 [100.0,100.0]	99.3 [95.3, 99.9]	94.3 [88.9, 97.1]	87.7 [80.5, 92.3]
SE	0.00000	0.00000	0.00673	0.01962	0.02933

Figure S3. Kaplan Meier curve for freedom from major adverse events over 24 months.

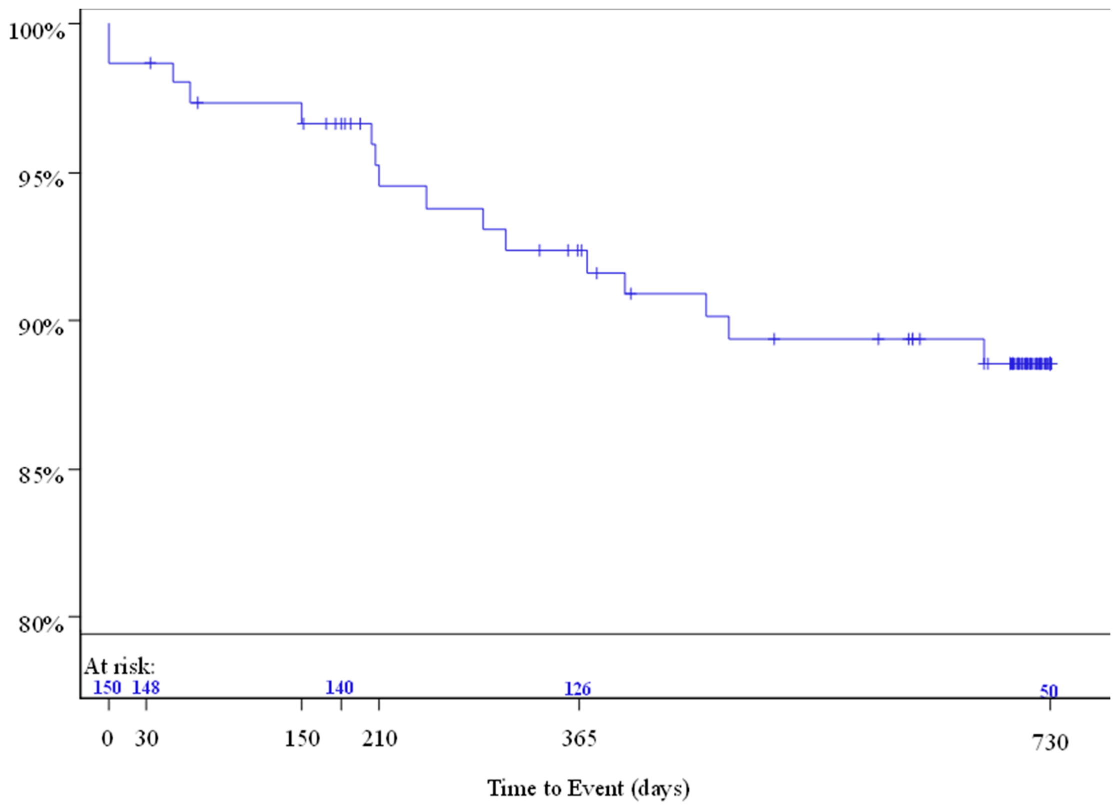

Description	Day 0	Day 30	Day 180	Day 365	Day 730
At risk	150	148	140	126	50
Events	2	2	5	11	16
Censored	0	0	5	13	86
Survival (estimate)	98.7 [94.8, 99.7]	98.7 [94.8, 99.7]	96.6 [92.1, 98.6]	92.4 [86.6, 95.7]	88.6 [82.0, 92.8]
SE	0.00937	0.00937	0.01474	0.02218	0.02696

Figure S4. Kaplan Meier curve for freedom from major adverse limb events over 24 months.

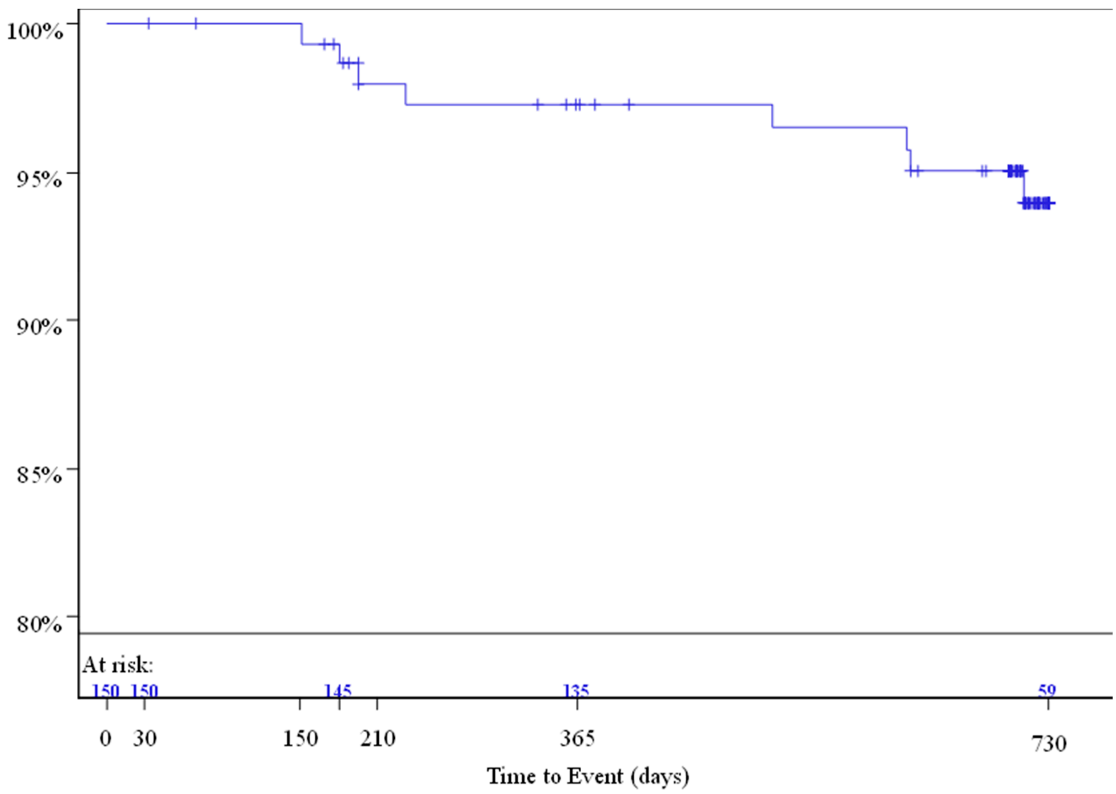

Description	Day 0	Day 30	Day 180	Day 365	Day 730
At risk	150	150	145	135	59
Events	0	0	1	4	8
Censored	0	0	4	11	85
Survival (estimate)	100.0 [100.0,100.0]	100.0 [100.0,100.0]	99.3 [95.3, 99.9]	97.2 [92.8, 99.0]	94.0 [88.2, 97.0]
SE	0.00000	0.00000	0.00673	0.01364	0.02102

Figure S5. Kaplan Meier curve for survival rate over 24 months.
